# Exosomes as Specific Vehicles for Delivery of Combination Therapies for Inhibiting Autophagy and Inducing Apoptosis in MYCN-Amplified Neuroblastoma Displaying Gut Dysbiosis: Current Challenges and Future Opportunities

**DOI:** 10.3390/brainsci16020125

**Published:** 2026-01-24

**Authors:** Kendall Leigh, Swapan K. Ray

**Affiliations:** Department of Pathology, Microbiology, and Immunology, University of South Carolina School of Medicine, 6439 Garners Ferry Road, Columbia, SC 29209, USA

**Keywords:** MYCN-amplified neuroblastoma, apoptosis, autophagy, engineered exosomes, combination therapies, gut dysbiosis

## Abstract

Neuroblastoma is a highly aggressive pediatric malignancy originating from neural crest progenitor cells, predominantly in the adrenal medulla. Amplification of the MYCN oncogene occurs in 20–30% of all neuroblastoma cases and approximately 50% of high-risk tumors, strongly correlating with poor prognosis, relapse, and multidrug resistance. MYCN-driven oncogenesis promotes tumor progression by suppressing apoptotic signaling and enhancing survival pathways, including autophagy—a key mechanism underlying resistance to chemotherapy and immunotherapy. This review examines current therapeutic strategies and resistance mechanisms in MYCN-amplified neuroblastoma, while introducing emerging approaches utilizing exosomes as precision drug delivery systems. Exosomes, nanoscale extracellular vesicles secreted by the tumor cells, exhibit natural tropism and can be engineered to selectively target neuroblastoma-specific biomarkers such as glypican-2 (GPC2), which is highly expressed in MYCN-amplified tumors. Leveraging this property, neuroblastoma-derived exosomes can be purified, modified, and loaded with small interfering RNA (siRNA) to silence MYCN expression, combined with chloroquine—an FDA-approved autophagy inhibitor—to simultaneously inhibit autophagy and induce apoptotic signaling. This dual-targeted approach aims to overcome drug resistance, reduce off-target toxicity, and enhance therapeutic efficacy through exosome-mediated specificity. Furthermore, gut dysbiosis has emerged as a critical factor influencing tumor progression and diminishing treatment efficacy in MYCN-amplified neuroblastoma. We propose integrating microbiota-derived exosomes engineered to deliver anti-inflammatory microRNAs (miRNAs) to the gut mucosa, restoring eubiosis and potentiating systemic anti-tumor responses. Collectively, exosome-based strategies represent a paradigm shift in formulating combination therapies, offering a multifaceted approach to target MYCN amplification, inhibit autophagy, induce apoptosis, and modulate the tumor-microbiome axis. These innovations hold significant promise for improving clinical outcomes in high-risk MYCN-amplified neuroblastoma patients.

## 1. Introduction

Neuroblastoma (NB) is the most common extracranial solid tumor in children [1]. It is considered an embryonic tumor because it originates in neural crest progenitor cells. It can be found anywhere along the sympathetic nervous system, but it is most often located on the adrenal glands. The clinical behavior of NB is highly heterogeneous, ranging from spontaneous regression to aggressive, metastatic disease with poor prognosis. The course of disease can be highly variable depending on the age of the child and the characteristics of the tumor itself [2]. Children diagnosed in infancy are more likely to have the low-risk NB classification, which can often spontaneously regress. With treatment often including surgery, these patients have an extremely high probability of full recovery [3]. Children diagnosed after the age of 18 months are more likely to have high-risk NB [4]. High-risk NB is characterized by aggressive growth and therapy resistance. Approximately half of all NB cases are classified as high-risk [5]. A critical molecular hallmark of high-risk NB is the amplification of the human myelocytomatosis neuroblastoma-derived (MYCN) oncogene, which is strongly correlated with the advanced disease, rapid progression, and significantly high resistance to conventional therapies, including chemotherapy and immunotherapy [1]. The structure of MYCN oncoprotein contains a basic helix-loop-helix (bHLH) domain, which allows it to bind to DNA and regulate gene expression. MYCN amplification or overexpression contributes to aggressive growth of NB. Current treatments for NB include immunotherapy, stem cell transplants, targeted therapies, radiotherapy, and chemotherapy [6]. NB is responsible for 15% of all pediatric cancer deaths [7]. Despite intensive multi-modal treatment strategies, the 5-year survival rate for high-risk NB patients remains distressingly low, underscoring an urgent need for novel therapeutic approaches that can effectively circumvent treatment resistance [8]. This article aims to explore the use of exosomes as target specific exciting nanovehicles for delivering potential combination treatments to the high-risk NB having MYCN amplification [9].

Therapy resistance in the high-risk MYCN-amplified NB is a complex and multifactorial phenomenon driven by both intrinsic molecular mechanisms and the intricate extrinsic interactions within the tumor microenvironment (TME). Factors contributing to therapy resistance may include enhanced drug efflux, alterations in drug metabolism, and activation of pro-survival signaling pathways. Fatefully, TME plays a significant role in fostering therapy resistance through bidirectional communication between NB cells and surrounding stromal and immune cells [10,11]. Recent studies have highlighted the indispensable role of extracellular vesicles (EVs), particularly exosomes, as key mediators of this intercellular communication, both promoting tumor progression and disseminating drug-resistant traits [9,12]. EVs are membranous structures derived from almost all cells, including NB cells. They consist of two major subtypes called microvesicles and exosomes. Microvesicles are formed from budding of the plasma membrane of the cell and tend to be larger than exosomes. Exosomes are derived from the plasma membrane of endosomes and can be much smaller [13]. Exosomes are nanosized (30–150 nm) lipid bilayer vesicles derived from the endosomal system and secreted by nearly all cell types, including NB cells. They act as ‘mini-messengers,’ carrying a diverse cargo of proteins, lipids, DNA, messenger RNA (mRNA), and microRNAs (miRNAs) from their parent cell to adjacent or distant recipient cells [14]. This cargo is protected from degradation in the extracellular space, and upon uptake by recipient cells, the exosomal contents can reprogram recipient cell functions, thereby modulating critical processes such as angiogenesis, immune evasion, metastasis, and drug resistance. Specifically in NB, tumor-derived exosomes have been shown to contain tumor-specific signatures, including oncogenic miRNAs that can horizontally be transferred to other cells, facilitating the acquisition of a drug-resistant phenotype [15,16].

**Figure 1:**
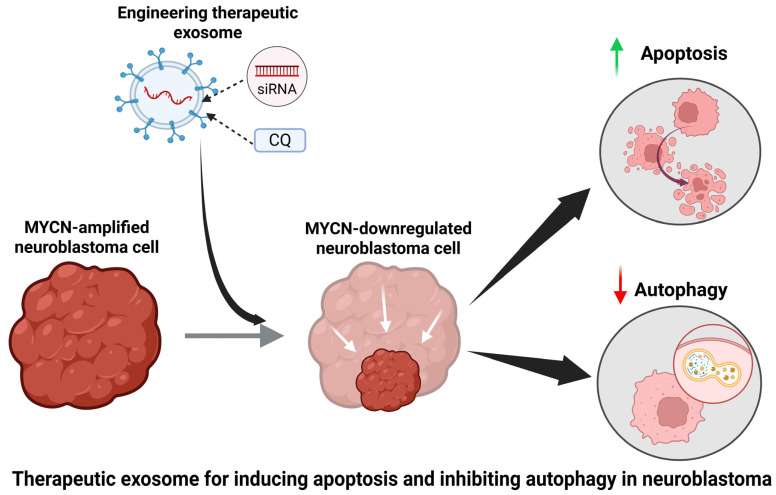
The diagram illustrates the use of therapeutic exosomes loaded with siRNA and chloroquine in the treatment of NB. Chloroquine is an autophagy inhibitor, and siRNA can be used to downregulate the expression of MYCN gene. The results of this treatment would be a reduction in autophagic activity and an increase in apoptotic activity in NB cells. Induction of apoptosis in NB cells is important to initiate a cell death mechanism without triggering inflammatory processes in surrounding tissue. Inhibition of autophagy is important because autophagy is a bulk degradation mechanism used by NB to contribute to therapy resistance. This figure was created using BioRender.

An effective treatment for NB cells would need to induce apoptosis in the tumor cells and inhibit autophagy concurrently. Apoptosis is a programmed cell death, which is the preferred method of killing cancer cells [17]. This is because apoptosis essentially causes cells to shrink and die as opposed to swelling and bursting. The rupture of cells can stimulate inflammatory reactions in nearby tissues [18]. Autophagy is a recycling mechanism used by cells to break down defective proteins or organelles that could be harmful to them. Cells use this process to maintain metabolic homeostasis [19]. This is an extremely beneficial mechanism in healthy cells, but it can introduce a major obstacle in controlling growth of cancer cells. Cancer cells can use autophagy to degrade proteins or drugs that can potentially be used to stop their proliferation [20]. Another key component of an effective cancer treatment would be specificity of the target. Treatments that accomplish the induction of apoptosis as well as the inhibition of autophagy must be specifically targeted to NB cells and avoid killing of healthy cells. The induction of death in healthy cells along with NB cells can lead to serious side effects. Exosomes derived from NB could theoretically serve as effective vehicles for selectively delivering one therapy aimed at inducing apoptosis and another therapy aimed at inhibiting autophagy in NB cells (Figure 1).

The very properties that allow exosomes to be drivers of oncogenic growth—their stability and capacity to transfer functional cargo to specific cells—at the same time make them attractive and promising natural nanocarriers for targeted drug delivery to overcome therapy resistance in high-risk NB [21,22]. Traditional drug delivery methods often face challenges such as poor bioavailability, systemic toxicity due to non-specific distribution, and inadequate tumor penetration. Exosomes offer an elegant solution by providing a biocompatible, low-immunogenic, and intrinsically targeted delivery system. Exosomes from tumor cells can be engineered and utilized as ‘Trojan horses’ to encapsulate and deliver various therapeutic agents, including conventional small-molecule chemotherapy drugs, miRNAs, or small interfering RNAs (siRNAs) designed to silence resistance-conferring genes or pathways, directly to the tumor site [23]. The potential of exosome-based therapy is particularly significant in the context of MYCN-amplified NB. The MYCN oncogene, while central to the disease, has proven difficult to directly target with conventional drugs. Exosomes could be engineered to deliver nucleic acid therapeutics (e.g., siRNAs or antisense oligonucleotides) specifically designed to suppress MYCN expression or downstream effectors involved in drug resistance [22,24]. Furthermore, the natural targeting capabilities of exosomes can be enhanced through surface engineering—modifying their surface proteins or lipids to display specific targeting moieties (e.g., antibodies or peptides) that bind to receptors overexpressed on MYCN-amplified NB cells. This strategy promises to significantly increase therapeutic efficacy while minimizing off-target effects on healthy tissues. Understanding the exosomal mechanisms that promote therapy resistance is opening new avenues for rational therapeutic interventions. Repurposing these endogenous nanocarriers to specifically deliver anti-resistance payloads holds immense promise for developing effective, targeted, and less toxic treatments that can finally overcome the therapy resistance associated with MYCN amplification and improve the devastating prognosis of this childhood cancer. Use of exosomes is the preferred method that investigators currently adopt because it provides a potential drug delivery system due to its small size and versatility of the contents that can be carried. The small size as well as the endogenous route of biogenesis allow exosomes to more easily travel. Exosomes also do tend to avoid triggering immune responses because they are not recognized as foreign objects by the immune system. They can carry proteins, nucleic acids, or small molecules as therapeutic agents [25]. These characteristics make exosomes an intriguing candidate for delivering therapeutic agents to treat MYCN-amplified NB.

## 2. Current Treatments for High-Risk NB and Urgent Need for Novel Approaches

Low-risk NB patients have a survival rate exceeding 95% with surgical resection of the tumor. The low-risk classification of the tumor results in no metastasis and very slim chances of recurrence [26]. On the other hand, high-risk NB patients have a much more complicated treatment plan with much lower success rates. High-risk NB is treated with high-dose chemotherapy, autologous stem cell transplantation, immunotherapies with anti-GD2 monoclonal antibodies, targeted therapies, or any combination of the above [27]. GD2 stands for ganglioside D2, a specific type of glycolipid found on the surface of cells of neuroectodermal origin. The letter “G” signifies a ganglioside, while “D” indicates two sialic acid residues, and “2” denotes its migration pattern in thin-layer chromatography. GD2 is widely considered a tumor-associated antigen because it is rarely expressed in normal tissues but highly expressed in various cancers including NB. Different treatment plans are needed for different subtypes of NB tumors because not all NB cells have the same genetic mutations. Approximately 20–30% of all NB cells share an amplification of the MYCN gene [28], which will be the target of the therapeutic agents using exosomes and focus of our further discussion in this article.

In the past, treatment for high-risk NB patients was like the approach for several cancers. Current methods of treatment for NB in the clinics come with some success as well as with some problems (Table 1). The focus was to increase the dose of chemotherapy as high as possible to increase patient survival rates. The myelotoxic effect of chemotherapy made this option less than ideal. To combat these effects, autologous stem cell transplantation was introduced. The idea was for the stem cells to decrease the side effects so that high doses of chemotherapy could continue to be used [29]. While this method was effective in increasing the dose of chemotherapy that was able to be used, survival rates did not significantly increase [30]. Event-free survival rates, meaning survival without relapse, remain between 30 and 40% using a combination of chemotherapy and autologous stem cell transplantation [31]. Any success with high-dose chemotherapy for NB, unfortunately, comes with side effects that make this approach concerning and controversial.

Immunotherapy is the use of therapeutic agents to boost the immune system defenses against malignant cells [32]. This has been the recent focus for treatment advancements for high-risk NB patients. Anaplastic lymphoma kinase (ALK) receptors are found on NB cells and are absent in normal tissues. ALK is one of the genes responsible for the oncogenic properties of NB. ALK inhibitors have been used to impair the growth of the tumor as well as to increase the expression of ALK receptors present on NB cell surface. ALK inhibitors are often used in combination with chimeric antigen receptor T (CAR T) cells to kill NB cells with minimal toxicity to the normal cells [33]. Another method is using antibodies to attack cancer cells. NB cells have a unique antigen called GD2 not found in most normal cells, as mentioned above. GD2 is expressed in neurons and peripheral pain fibers as well [34]. Anti-GD2 monoclonal antibodies have been used to target NB cells. These antibodies exert their effect using multiple mechanisms. Anti-GD2 triggers complement lysis of NB cells, which can be responsible for the severe pain that patients experience due to treatments. They also activate natural killer cells as well as phagocytosis of NB cells [35]. The advantage of immunotherapy for NB is that it is not associated with long term toxicity like several chemotherapeutic agents [36].

**Table 1:** Currently used common methods of treatment for NB in clinical practice show some success and some problems.

Method	Process	Outcomes	Problems	References
Chemotherapy	Drugs used to induce apoptosis often used in conjunction with autologous stem cell transplantation	Show 30–40% event-free survival rates	Non-specific to tumor cells, so patients experience severe myelotoxic effects	[29,30,31]
Autologous stem cell transplantation	Often used in conjunction with chemotherapy to replace healthy cells lost by high-dose chemotherapy	Do not significantly improve outcomes above numbers with only chemotherapy	Allowed for a higher dose of chemotherapy to be tolerated by patients but do not improve survival rates	[29,30,31]
ALK Inhibitors with CAR T cells	ALK inhibitors increase the expression of ALK on surface of NB cells that can be targeted using CAR T cells to induce apoptosis	Successfully targets ALK mutated tumors specifically so that there are limited side effects	Does increase expression of ALK in tumors with low ALK density to improve efficacy but often not enough to be sufficient in low ALK density tumors	[33]
Anti-GD2 monoclonal antibodies	Antibodies are used to target the GD2 receptor found on most NB and can activate lysis of NB cells and NK cells to induce apoptosis	Outcomes show improvements in 40–50% cases	Often relapsed tumors become resistant to this treatment and cause severe pain due to presence of GD2 receptors on nerve cells	[34,35]

The most success in NB therapy has been found using multi-modal treatments. The current standard is a combination of myeloablative therapy (high doses of chemotherapy and or radiation therapy) coupled with stem cell transplantation followed by anti-GD2 immunotherapy [37]. Despite the intensive treatment regimen, the survival rate remains around 50% for high-risk NB. Most of the deaths attributed to NB patients are from relapsed disease post-treatment. Residual malignant cells develop resistance to therapy, which results in an inability to treat [38]. This is why advancements in treatment strategies are so imperative, it can not only avoid the drug resistance mechanisms of NB but also improve the treatment outcomes.

The use of exosomes as a precision drug delivery vehicle has the potential to evade known NB mechanisms of drug resistance. Currently exosomes have been used in NB as a prognostic tool. Exosomes derived from NB cells carry proteins that can give information on the phenotypic characteristics of the tumor, which is useful in determining prognosis and treatment options [39]. Other studies have focused on targeting NB-derived exosomes to inhibit NB cell-to-cell communication and target signaling pathways initiated by the exosomes [40]. Research in this area has been focused on the role of exosomal proteins in the growth and modulation of the TME. The changes to the TME cause the disruption to the immune system, allowing the tumor cells to proliferate [24]. The next step would be harnessing the ability of NB-derived exosomes to communicate with NB cells and use them as a specific vehicle for delivering therapeutic agents to induce apoptosis in the NB cells.

## 3. Drug Resistance in MYCN-Amplified NB and New Therapeutic Opportunities

The biggest obstacle in NB treatment is the prevalence of multiple drug resistance mechanisms in high-risk tumors. The heterogeneity of NB coupled with adaptability of the tumor for survival and growth poses extra challenges. NB can resist all known methods of treatments including chemotherapy, radiation therapy, targeted therapies, and immunotherapy [41]. NB utilizes a multitude of pathways to master highly effective therapy resistance. These mechanisms include ATP-binding cassette (ABC) transporter proteins, autophagy, differentiation of adrenergic cells to mesenchymal fibroblasts, antibody internalization, and modulation of the TME [42].

The mechanisms used in chemotherapy resistance include ABC transporters and autophagy. ABC transporter proteins are a family of membrane transporters. ABCB subfamily members are commonly found at blood organ barriers. ABCB1 is also known as multidrug resistance protein 1 (MRP1) and is used by several drug-resistant cancers to evade chemotherapy treatments [43]. MRP1 is expressed by NB cells, allowing them to export a range of chemotherapies. However, MRP1 is not specific to NB and is found in several locations in the body including the blood–brain barrier, gastrointestinal tract, liver, and kidneys. While eliminating MRP1 does increase the efficacy of chemotherapy on NB cells, it also has severe side effects because of how prevalent it is in normal tissues for their normal functions [44]. Autophagy is a recycling process for cellular homeostasis in which a cell degrades its damaged organelles or cytosolic proteins and drugs. Cancer cells are highly proficient in using autophagy to overcome chemotherapy by eliminating organelles damaged by the drug or eliminating the drug itself [45].

The mechanisms used by NB in conferring immunotherapy resistance include epigenetic regulation of cell types, antibody internalization, and modulation of the TME. Anti-GD2 antibodies have been successful in treating high-risk NB, but approximately half of these patients show relapse of the disease [46]. Relapsed NB is often resistant to anti-GD2 immunotherapy. NB has two major cell types, adrenergic and mesenchymal. Relapsed tumors often have a higher proportion of mesenchymal cells, which have much lower expression of GD2 making anti-GD2 immunotherapy ineffective [47]. NB cells are capable of converting between the two cell types via epigenetic regulation based on the environment they are in to increase their chances of survival in the challenging TME. The adrenergic cell type is associated with increased proliferation and tumor growth. Tumors of this cell type tend to respond well to anti-GD2 immunotherapy due to high expression of GD2 on the cell surface. Under normal conditions, adrenergic cells are more common because of the more aggressive growth abilities. In times of stress, including undergoing immunotherapy, NB cells transition to the mesenchymal cell type to promote survival and often the reason for recurrent tumors to become resistant to anti-GD2 immunotherapy [48]. Antibody internalization of the anti-GD2 antibodies is another mechanism of therapy resistance. NB cells are often capable of internalizing the target complexes for anti-GD2 antibodies to reduce the cell surface expression of GD2. This results in a reduction in the amount of bound monoclonal antibodies to NB cells, decreasing the effectiveness of the treatment [49].

**Figure 2:**
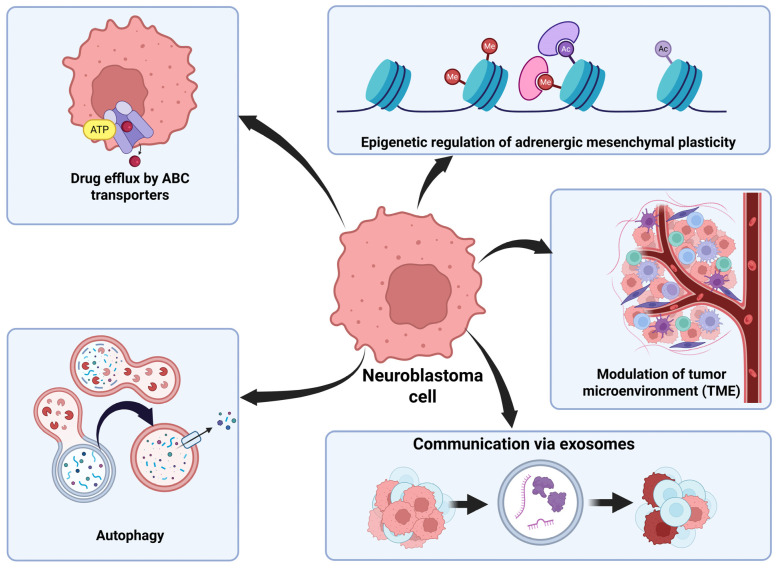
NB has several mechanisms of drug resistance. ABC transporters are used to export chemotherapeutic agents from cells to avoid their damage. Autophagy is a process used to degrade damaged organelles or harmful proteins for efficient recycling to increase cell survival in strenuous conditions. Epigenetic regulation allows for NB cells to transition between adrenergic and mesenchymal cell types with different properties depending on the characteristics of the tumor environment. NB cells can modify the TME by secreting factors to inhibit immune cells. NB cells use exosomes to communicate with surrounding cells and transfer oncogenic material between cells. This figure was created using BioRender.

The TME is made up of a combination of the extracellular matrix and surrounding tumor cells, vascular endothelial cells, mesenchymal stromal cells, fibroblasts, and immune cells [50]. NB cells have an especially immunosuppressive TME, which causes difficulty in developing effective immunotherapies. Tumor-associated macrophages found in the TME secrete the cytokine interleukin-6 (IL-6) to enhance communication between the tumor cells and vascular endothelial growth factors to promote proliferation of the tumor cells as well as to promote angiogenesis (formation of new blood vessels) to supply nutrients to the tumor. These macrophages also inhibit the body’s immune cells, which can cause resistance to immune checkpoint inhibitors, which block several proteins such as programmed death receptor-1 (PD-1), programmed death receptor ligand-1 (PD-L1), and cytotoxic T lymphocyte-associated antigen-4 (CTLA-4), which are normally known to prevent T cells from killing cancer cells [51]. NB cells themselves can also secrete immunoregulatory mediators, which can reduce infiltration of immune cells into the tumor and decrease effectiveness of immunotherapies [52]. Overall, drug resistance mechanisms pose huge challenges in the treatment of NB (Figure 2).

The increased expression of MYCN fundamentally remodels the NB cells, conferring a highly complex multi-faceted drug-resistant phenotype. The genomic event of MYCN amplification drives aggressiveness of this tumor by promoting cell proliferation, inhibiting cell differentiation, and especially being addicted to autophagy for therapy resistance to conventional chemotherapeutic agents, presenting a formidable barrier to its successful treatment and thus a major focus of current research efforts in the NB field [53,54]. The multi-faceted drug resistance phenotype in MYCN-amplified NB can be broadly categorized into several key biological processes. First, evasion of apoptosis is one of the core mechanisms of chemoresistance conferred by MYCN. While MYCN is known to induce both proliferation and apoptosis, its overexpression in the context of NB is known to disrupt the delicate equilibrium in favor of survival [54]. Because it is a transcription factor, MYCN regulates the transcription of several genes involved in apoptosis, notably increasing the expression of the negative p53 regulator murine double minute 2 (MDM2). MDM2 in turn promotes MYCN stability, creating a positive feedback loop that blocks the pro-apoptotic functions of p53. Furthermore, MYCN-amplified cells can resist treatment only when there is additional dysfunction in the apoptotic pathways, such as caspase deficiency [54]. Second, drug efflux enhancement due to MYCN amplification promotes multidrug resistance phenotype by modulating the expression of ABC transporter genes, which function as drug efflux pumps to actively transport chemotherapeutic agents out of the cancer cells, thereby reducing the intracellular drug concentration below cytotoxic levels. This mechanism is a general and widespread contributor to chemotherapy resistance in MYCN-amplified tumors [55]. Third, metabolic reprogramming, which is a hallmark of cancer that contributes to survival and drug resistance, results from MYCN amplification [53,56]. Overexpression of MYCN drives a shift in metabolism, promoting aerobic glycolysis (the Warburg effect) and, more importantly, altering lipid metabolism. Specifically, MYCN amplification has been shown to upregulate the fatty acid transport protein 2 (FATP2), a molecule that mediates the cellular uptake and biosynthesis of fatty acids [57]. This reliance on fatty acids for energy and growth creates a metabolic dependency that is unique to MYCN-amplified tumors and contributes to their aggressiveness and treatment resistance [58]. Fourth, cellular state and epigenetic alterations are caused by MYCN overexpression. MYCN-amplified NB cells change cellular state from a mesenchymal to a more aggressive and undifferentiated adrenergic state, which is associated with increased proliferation and poor prognosis. This state transition is mediated by MYCN promoting the expression of epigenetic regulators [59]. Additionally, other epigenetic and non-coding RNA mechanisms, such as miRNAs and long non-coding RNAs (lncRNAs), have been shown to be deregulated by MYCN and contribute to the acquisition of chemoresistance [60].

Tumor cells can utilize extrachromosomal DNA (ecDNA), a circular form of DNA different from chromosomes, to amplify oncogenes such as MYCN to exceptionally high copy numbers through an unusual inheritance model of ecDNA [61]. The lack of centromeres in the structure of ecDNA molecules permits tumor cells harboring ecDNA transmission of their ecDNA copies unequally to their descendant cells during cell division, causing rapid accumulation of ecDNA copies. This uneven separation of ecDNA copies serves as a mechanism to promote tumor heterogeneity at genetic level [62]. This ecDNA discrepancy makes the tumor much more complex and generally limits the effectiveness of treatment, resulting in therapy resistance. A recent study used NB cell lines, patient-derived xenografts, and patient tumor samples to show how ecDNA driven MYCN copy-number heterogeneity created therapy resistance [63]. This study highlighted how ecDNA modulated MYCN dosage, causing phenotypic and molecular heterogeneity and determined tumor cell fate under therapy. The NB cells with high copies of MYCN and harboring ecDNA (MYCN ecDNA) showed high proliferation, replication stress, and DNA damage. Given the limited efficacy of conventional chemotherapy in the NB having MYCN amplification and deregulation, investigators are aggressively pursuing novel and targeted strategies aimed at disrupting oncogenic activities of MYCN, either directly or indirectly, to resensitize tumor cells to treatment [54]. Moreover, novel drug delivery systems such as exosomes in conjunction with innovative combination therapeutic strategies are being developed to overcome MYCN-driven drug efflux and improve the efficacy of chemotherapeutic drugs for induction of apoptosis and inhibition of autophagy in the MYCN-amplified NB.

Exosomes can also play an integral part in the evasion of immunotherapy in NB. Tumor-derived exosomes are released from NB cells to inhibit T cells and natural killer cells [64]. Exosomes are also capable of exchanging miRNAs between NB cells and surrounding monocytes. NB cells can receive miR-155, which is an oncogenic miRNA, from nearby cells. High levels of miR-155 are used to target an inhibitor of telomerase, the enzyme that maintains telomeric length at the chromosomal ends and helps tumor cells sustain DNA replication and cell proliferation. An increase in telomerase activity is associated with drug resistance in the tumor and poor prognosis of the patients [15]. Extracellular vesicles including exosomes are also responsible for transferring drug-exporter proteins from drug-resistant cells to non-resistant cells, as well as binding monoclonal antibodies in circulation to prevent them from entering the tumor [65]. Interestingly, the ability of exosomes to effectively communicate with NB cells can be turned around to use them against the tumor itself. Artificially loading NB-derived exosomes with pro-apoptotic and anti-autophagic factors is a way to bypass the various drug resistance mechanisms. NB cells would recognize the exosomes, so the exosomes could then infiltrate into the tumor and deliver the therapies that otherwise were not able to obtain access to the NB cells.

## 4. Cell Surface Receptors as Unique Biomarkers and Therapeutic Targets in NB

Targeting therapies to induce apoptosis and inhibit proliferation of NB cells is an important step in the development of a successful NB treatment strategy. The next obstacle is identifying biomarkers that are unique in NB cells and not present in normal tissues to minimize toxicity to the normal cells. Treatments utilizing targets common to both NB and healthy cells cause severe side effects due to damage caused to normal surrounding tissues. There are two different types of toxicity called on-target and off-target. On-target toxicity refers to the drug acting on its intended target protein, but in both healthy and malignant cells. Off-target toxicity occurs when the drug acts on unintended targets as well [66]. NB cells have been notoriously difficult to identify a suitable unique target in them. One of the reasons for this is the heterogeneity of NB cells [67]. There are multiple gene mutations associated with NB but there are no mutations currently identified in all high-risk tumors. Some unique biomarkers such as ALK, protein tyrosine phosphatase non-receptor type 11 (PTPN11), alpha-thalassemia intellectual disability—X-linked syndrome (ATRX), and MYCN are the most commonly found in high-risk NB patients [68]. MYCN is the most common oncogene amplification but even so is only found in an estimated 20–30% of all NB tumors and 50% of high-risk patients [69]. There are also chromosomal aberrations associated with poor prognosis of NB patients including 1p and 11q deletions as well as 17q gain, but these are also not present in every high-risk tumor [70]. Another aspect of this heterogeneity is the plasticity of NB cells to convert between adrenergic cells and mesenchymal cells [71]. These two cell types have different levels of gene expression and respond differently to treatment [72].

Identifying a target on the cell surface, which is widely expressed by NB cells and has little to no expression in normal tissue, is important for an exosome-based drug delivery approach to make the treatment as specific as possible [66]. Tropomyosin receptor kinase B (TrkB), which is a receptor for brain-derived neurotrophic factor, plays crucial roles in the development, survival, and function of the nervous system cells. B7-H3 (CD276), a member of the B7 family of proteins, is a key player in NB progression. This is an immune checkpoint molecule selectively expressed in tumor cells and immune cells in the TME. Glypican 2 (GPC2) is a cell surface proteoglycan involved in cell signaling pathways for influencing cell growth and tissue development. Some potential therapeutic targets in NB include GD2, ALK, TrkB, B7-H3, or GPC2 [73,74,75]. GD2 has been established as a highly effective target that is currently used in immunotherapy due to its high level of expression in most NB cells, but it is associated with severe adverse effects due to its expression in peripheral nerve cells as well [76]. It is also highly characteristic for relapsed tumors to become resistant due to the low expression of GD2 in mesenchymal-type NB cells [77]. ALK is a receptor tyrosine kinase that has been identified as a viable target for NB therapy, but it is not consistently expressed by NB cells making it only effective in ALK-mutated tumors, which is only 10.9% of MYCN-amplified tumors [78]. TrkB is a receptor that is highly expressed in high-risk NB cells, but it is also expressed in other areas that may cause neurotoxic effects if it is used as a therapeutic target due to the expression of TrkB in the central nervous system cells [79,80]. B7-H3 is highly expressed in NB cells and has low levels of expression in normal tissues so there would be limited toxicity. It is notable that B7-H3 expression remains high in NB patients with low levels of GD2 after anti-GD2 therapy [81]. GPC2 is a receptor that is highly expressed in MYCN-amplified tumors. It is silenced in normal tissues, making it an ideal therapeutic target in NB [82]. It would be the best target in terms of working on the highest proportion of tumors and limiting adverse effects. Cell surface receptors are promising therapeutic targets in NB (Table 2). Ongoing trials are taking place in various stages regarding the efficacy of these targets. While GPC2 has not yet been targeted in clinical settings, there are promising results in some in vivo studies to demonstrate its potential as a target for NB treatments.

NB-derived exosomes represent a promising and natural nanovehicle for specifically targeting NB. These nanovehicles are recognized by the tumor because they originate from it and share similar surface receptors, a phenomenon known as natural tropism [90]. However, natural tropism alone may not guarantee sufficient uptake of exosomes by the tumor cells [91]. To enhance uptake, additional active targeting using a cell surface receptor is necessary [92]. Based on the evidence above, the GPC2 receptor stands out as the most favorable target due to its high level of expression in MYCN-amplified NB and negligible presence in normal tissues.

Isolating exosomes from the tumor cells followed by purifying them to eliminate oncogenic contents is essential to prevent the possibility of promoting further tumor growth [93]. The benefits of using NB-derived exosomes would be high biocompatibility and low immunogenicity allowing the exosome to carry the therapeutic agents to the tumor without interference. The specific targeting of these exosomes to NB cells minimizes adverse effects. The disadvantage of this method is the possibility for the exosome to be carrying residual oncogenic cargo after purification and loading, which potentially creates risks of tumorigenesis. An alternative strategy would be the employment of mesenchymal cell-derived exosomes that eliminate the risk of tumorigenesis but sacrifice some of the high specificity to NB cells [94]. These exosomes can then be loaded with pro-apoptotic and anti-autophagic therapeutic agents. The contents of these exosomes will then be delivered to the tumor for promoting apoptotic cell death in the NB cells. The specific cell surface target will reduce the delivery of therapeutic agents to surrounding monocytes that do not have a GPC2 receptor. Purification of the exosome from oncogenic contents will result in reduced immunosuppression but surface proteins can still cause residual immunosuppression [95].

## 5. Targeting Apoptosis-Blocking Factors in NB for Induction of Apoptosis

Apoptosis is a genetically controlled cell death mechanism. Apoptotic cell death can occur through two major pathways called extrinsic or receptor-mediated pathway and intrinsic or mitochondria-mediated pathway in NB cells in vitro and in vivo [96,97]. The extrinsic pathway is triggered by ligands binding to death receptors on the cell surface. When bound, these receptors signal activation of the cysteine protease caspase-8. Then, caspase-8 activates caspase-3, which is the final executioner cysteine protease committed to take the process of apoptosis to the end [98]. The intrinsic pathway is activated by stress signals coming from inside the cell, including damage to DNA. Expression of the Bcl-2 homology 3 (BH3)-only proteins is upregulated because of these stress signals. BH3-only proteins such as Bax and Bak are pro-apoptotic and promote downstream steps of apoptosis. These proteins disrupt the outer mitochondrial membrane to allow release of mitochondrial cytochrome c into the cytoplasm. Cytochrome c binds to apoptotic protease-activating factor 1 (Apaf 1) to form the apoptosome. The apoptosome activates caspase-9, which then activates caspase-3. This is where both the extrinsic and intrinsic pathways merge [99]. NB cells can evade apoptosis inducing signals by silencing caspase-8 via MDM2 gene that is a negative regulator of tumor suppressor gene p53, activation of the phosphoinositide 3-kinase (PI3K)/protein kinase B or Akt/mechanistic target of rapamycin (mTOR) pathway, and overexpression of survivin and other anti-apoptotic proteins of the Bcl-2 family [100].

In order to induce apoptosis in NB tumors, these survival mechanisms must be bypassed. This has been difficult to accomplish because of the numerous ways NB cells can disrupt apoptotic signals. MDM2 is often overexpressed in NB while p53 is typically mutated despite low activity in NB cells, because MDM2 is a negative regulator of the tumor suppressor gene p53, giving survival advantage to the NB cells. However, an MDM2 inhibitor can be used to increase the expression of p53 to promote apoptosis in NB cells. This approach has shown success in preclinical phases by shrinking NB with low off-target toxicity [101]. The PI3K/Akt/mTOR pathway is triggered by the ALK receptor and TrkB [102]. This pathway can block apoptosis via inhibiting pro-apoptotic genes including p53 and activating the mTOR complex that plays a key role in cell proliferation [103]. Inhibiting transcription of survivin (a potent anti-apoptotic protein) can be an effective strategy because survivin is present in most of the high-risk NB tumors and it inhibits apoptosis via blocking caspases [104,105]. Besides, Bcl-2 itself is a prominent anti-apoptotic protein present in most NB tumors. The induced myeloid leukemia cell differentiation protein, also known as Mcl-1, is another anti-apoptotic protein in the Bcl-2 family and present in many high-risk NB patients. Inhibitors of these factors allow pro-apoptotic members of the family, such as Bax and Bak, to initiate release of mitochondrial cytochrome c into the cytoplasm for activation of the downstream caspases [106,107].

**Figure 3:**
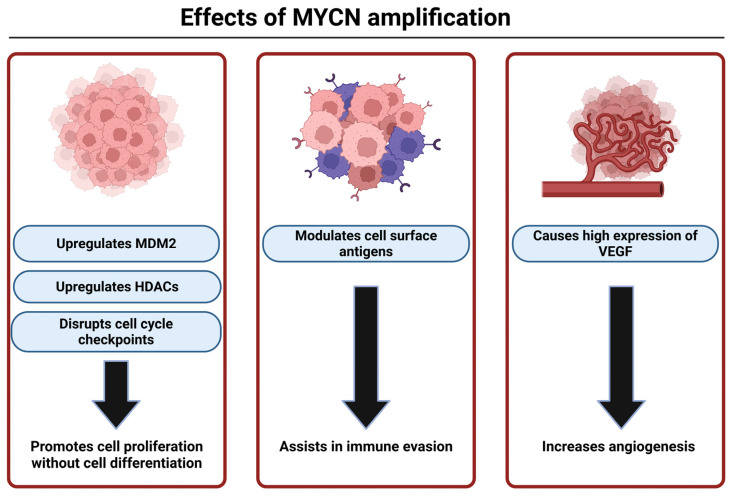
MYCN plays multiple roles in NB development, growth, and survival. MYCN-amplified tumors are notoriously aggressive because of the various ways that MYCN can use to increase cell survival and evade efficacy of treatments. This figure was created with BioRender.

As mentioned earlier, MYCN is the oncogene mostly found to be associated with high-risk NB. It is present in an estimated 20–30% of all NB and 50% of high-risk NB [9]. MDM2 is upregulated by MYCN, which leads to inhibition of p53 allowing for increased cell proliferation in the tumor [58]. MYCN can also promote tumor growth by disrupting the cell cycle checkpoints causing uncontrolled cell proliferation. It can modulate the expression of the antigens on the NB cell surface, helping it with immune evasion. MYCN amplification is also associated with high-level activation of the PI3K/Akt/mTOR pathway, which produces vascular endothelial growth factor (VEGF) for angiogenesis that assists in tumor survival. MYCN also upregulates histone deacetylases (HDACs) to inhibit differentiation and silence tumor suppressor genes [108]. Overall, MYCN plays a significant role in fostering pro-survival mechanisms in MYCN-amplified NB (Figure 3).

Therefore, MYCN is an ideal therapeutic target in the MYCN-amplified NB tumors, as it plays a variety of roles in maintaining the survival mechanisms in NB [109]; but it has been notoriously difficult to effectively target in the past due to its role as a transcription factor for multiple genes [110]. There has been success in using siRNA technology to silence the MYCN gene for induction of apoptosis [111]. Loading the NB targeted exosomes with siRNA to silence MYCN would increase efficacy by more efficient delivery to the NB cells and will have limited off-target effects due to the low to negligible expression of MYCN in normal cells after birth [112].

## 6. Autophagy as an Adversary in Strategies for Controlling Growth of MYCN-Amplified NB

Autophagy, also known as self-eating, is a bulk recycling mechanism used to maintain cellular homeostasis and is a beneficial mechanism in healthy cells. It can be either pro-cell death or anti-cell death depending on the needs of the cell [113]. MYCN-amplified NB cells often use autophagy as a mechanism of drug resistance [114]. These NB cells can degrade organelles damaged from chemotherapy or pro-apoptotic factors to continue growth [115]. Many treatments that have been used for NB intended to induce apoptosis can also induce autophagy, which ultimately reduces their therapeutic efficacy. Autophagy can also delay cell death in MYCN-amplified NB cells that have had the MYCN gene knocked down. In MYCN-amplified NB cells, autophagy can act as a pro-survival mechanism, delaying cell death when the MYCN gene is knocked down or inhibited. While MYCN knockdown is a promising therapeutic strategy, the pro-survival autophagy can compromise its effectiveness. This results from inactivation of the PI3K/Akt/mTOR pathway leading to a decrease in the activation of the mTOR complex 1 (mTORC1), which is normally upregulated by MYCN in the MYCN-amplified NB [116].

**Figure 4:**
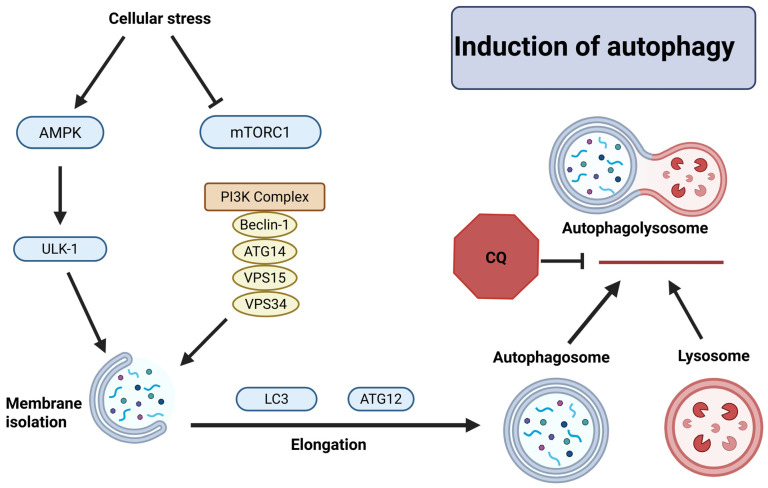
Cellular stress causing induction of autophagy in NB cells. AMPK phosphorylates ULK-1, which alongside the PI3K complex forms the isolation membrane. The membrane is expanded by LC3 and ATG12 to form the autophagosome. The autophagosome fuses with the lysosome to begin degradation of autophagosome contents. Chloroquine (CQ) is a known autophagy inhibitor that targets the fusion of the autophagosome to lysosome. This figure was created with BioRender.

The autophagy pathway is triggered by MYCN knockdown, chemotherapy, or stress signals from the cells [117]. Any one of these events activates adenosine monophosphate-activated protein kinase (AMPK) and inhibits mTORC1 (Figure 4) [118]. The initiation phase of autophagy includes phosphorylation of UNC-51-like kinase 1 (ULK-1) complex to induce nucleation. In this process, the class III phosphoinositide 3-kinase (PI3K) complex containing Beclin-1, autophagy-related gene 14 (ATG14), vacuolar protein sorting 15 (VPS15), and VPS34 are phosphorylated to form the isolation membrane. This complex generates phosphatidylinositol 3-phosphate (PI3P) to recruit ATG proteins to the site of formation of isolation membrane. Microtubule-associated protein light chain 3 (LC3) and ATG12 complex are recruited to elongate the membrane. The ATG12 complex closes the membrane and forms a complete autophagosome. The autophagosome fuses with lysosome to form autophagolysosome and then lysosomal enzymes digest its contents (peptides, nucleic acids, lipids, and carbohydrates) [119,120].

Inhibiting autophagy in the MYCN-amplified NB is an important therapeutic strategy to promote more effective way of inducing apoptosis, as autophagy can be used as an alternative survival mechanism after MYCN is no longer present in the tumor cells [121]. There are several protein complexes that have potential to be targeted for autophagy inhibition including ULK-1, VPS34, and Beclin-1. These options are currently being explored but are still in preclinical stages [122]. Chloroquine (CQ) and hydroxychloroquine (HCQ) are currently the only two drugs approved by the United States Food and Drug Administration (FDA) for autophagy inhibition. They both work by interrupting formation of autophagolysosome and impairing degradation abilities of lysosomes by modifying cellular pH [123]. While CQ is successful in inhibiting autophagy and enhancing efficacy of cancer treatment, it also has harsh side effects in other tissues. Autophagy is used by multiple organs including neurons, liver, and heart, but it is especially important for the function of kidneys. The inhibition of autophagy coupled with chemotherapeutic drugs can result in severe kidney damage depending on the dose and duration of use of the autophagy inhibition [124]. Therefore, using NB-specific exosomes to deliver CQ to NB would reduce the adverse effects by increasing the specificity of the target. Delivery via engineered exosome will allow for less toxicity to the essential organs by minimizing exposure of healthy cells to the drug.

## 7. Extracellular Vehicles as Potential Drug Delivery Systems

Extracellular vesicles (EVs) with a lipid bilayer membrane are nanosized vesicles that are released with varying contents from virtually all cell types. EVs are classified into subtypes based on size and method of formation. These include apoptotic bodies, oncosomes, microvesicles, and exosomes [125]. Apoptotic bodies are a specific type of EV that is released from cells during the last stage of apoptosis. They often contain remnants of organelles, proteins, and DNA and are thought to be functional in immune modulation and tissue regeneration [126]. Oncosomes are large vesicles released from malignant cells and carry oncogenic cargo thought to be used in transferring material to non-malignant cells [127]. Microvesicles are formed via the outward budding of the plasma membrane of cells. They are then pinched off and released into the extracellular space [128]. The release of microvesicles occurs at a basal rate in most cells but is greatly increased in stressful environments including cell injury and inflammation. They range from 50 to 1000 nm in size and are key components of communication between cells, angiogenesis, and immune response [129]. Exosomes at 30–150 nm diameter are the smallest of all the EV subtypes. They are formed by the inward budding of the endosome membrane to form multivesicular bodies that then fuse with the plasma membrane of the cell to release the exosomes into the extracellular space [130]. Exosomes are primarily important in intercellular communication and are used for transfering proteins, RNA, and lipids to the surrounding cells. They are also used by cancer cells to regulate TME, promote progression of tumor growth, and suppress the immune system activity [131].

Exosomes and microvesicles function similarly in their roles in cellular communication [132]. There is ongoing research involving the therapeutic application of each type. While the larger size of microvesicles compared to exosomes allows for the loading of larger cargo, it makes them more difficult to be taken up by the target cells. Relatively, the lack of standardization in production of microvesicles also makes them more difficult to reproduce, which is the main reasoning for the preference of exosomes in treatment developments [133]. Exosomes are currently used as diagnostic and prognostic tools in many types of cancer due to their high specificity in tumor biomarkers [134]. Exosomes are being explored as an option for targeted therapy in cancer due to their natural communication role, biocompatibility, and ability to travel in circulation without triggering an adverse immune response [135].

Current research in the realm of EVs is rapidly evolving. Exosomes are now able to be isolated in laboratory settings and modified for potential therapeutic applications [136]. The use of NB cell-derived exosomes with appropriate modification would allow for efficient delivery of custom-designed combination therapies to the tumor while limiting side effects. This is due to the ability of exosomes to specifically target tumor cells and deliver treatment to induce apoptosis and inhibit drug-resistant mechanisms such as autophagy while simultaneously avoiding immune system interference.

## 8. Exosomes as the Most Effective Drug Delivery Vehicles

**Figure 5:**
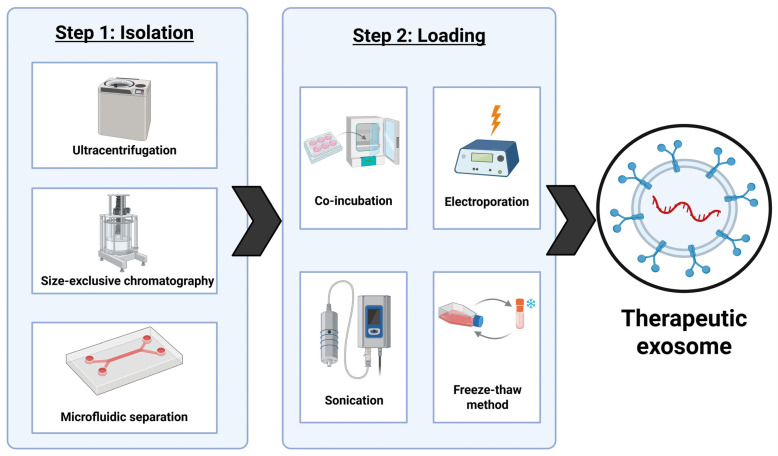
The schematic presentation of different methods of preparing exosomes for therapeutic use. This figure was created using BioRender.

Exosomes have potential to be more effective than any synthetically derived carriers because several disadvantages of synthetic drug carriers can be solved by biological properties of exosomes for therapeutic use. Their endogenous route of formation allows for them to evade immune detection while synthetic carriers have potential for inducing unwanted toxicity [137]. Exosomes are small nanocarriers that help them pass through membranes more efficiently than larger vesicles [138]. They also benefit from both natural tropism (directional movement in response to an environmental stimulus) as well as active targeting techniques to make them tumor specific [139]. However, a drawback of using exosomes in delivering therapeutics is that they are currently expensive to engineer with current methods giving relatively low yields of quality exosomes for therapeutic uses [140]. Exosomes must be isolated from parent cell, purified, and loaded with therapeutic RNAs, proteins, or drugs. Some isolation techniques include ultracentrifugation, size-exclusive chromatography, and microfluidic separation (Figure 5) [141].

Ultracentrifugation is a common method of isolation for large samples, but it risks structural damage to the exosomes along with high cost and time consumption. Size exclusion chromatography is much more cost efficient and faster, but it can compromise the purity of the batch of exosomes. Microfluidic methods are rapid and have the highest level of purity but require expensive equipment [142]. Currently, all methods of isolation of exosomes are both time consuming and costly, which is one of the biggest obstacles for the translation of this research into the clinical stages. The loading process can be completed at pre-secretion of the exosomes from the cell or post-isolation of the exosomes. Pre-secretion methods entail transfecting the parent cells with RNA that can then be packaged into exosomes to be released. A pre-selection method is not preferred due to its inability to track loading efficiency [143]. Post-isolation methods include co-incubation, electroporation, sonication, and the freeze–thaw. Co-incubation is not disruptive to the exosome membrane but as a result, it is only effective for small hydrophobic molecules. Both electroporation and sonication are methods that increase membrane permeability for small hydrophilic molecules including RNA and proteins, but both risk compromising integrity of the exosome membrane. The freeze–thaw method is effective for loading drugs into exosomes, but it must be performed in careful conditions to avoid damaging the exosomes beyond use [144]. There are also no high-yield methods of loading the exosomes, which is another obstacle to large-scale production and clinical implementation of this research. Any process is expensive to produce loaded exosomes, providing a relatively low percentage of pure and usable exosomes. This process would need to be streamlined and made cost-effective for exosomes to replace current therapeutic techniques.

Exosomes are currently being explored as potentially effective drug carriers for use in cancer treatments. Exosome-based therapy is still in early stages of development [145]. There are several ongoing studies in preclinical phases that are showing promising results for successful use of exosomes to deliver both chemotherapeutic drugs and miRNA or siRNA therapeutics (Table 3). Combination therapy utilizing exosomes as natural nanocarriers is also being explored. Loading the exosomes with two different therapeutic agents allows for creating its ability to target multiple resistance mechanisms that the tumor cell uses for survival [146]. An important aspect of combination therapy is evaluating the impact that the agents loaded together have on each other. Ensuring a synergistic effect will enhance the effectiveness of the treatment [147]. There are ongoing trials for the use of combination therapy in exosomes, but none of these has yet been successfully applied to NB treatment. However, promising results in early trials for other cancers are promoting the idea that these methods are worth exploring in the future for NB treatment as well. A combination therapy would be ideal for NB treatment because of the complex survival and drug resistance mechanisms present in this tumor.

The preclinical trial utilizing A15-Exosomes (A 15-Exos) produced from monocyte-derived macrophages loaded with doxorubicin and cholesterol modified miRNA-159 to treat triple-negative breast cancer mentioned successful inhibition of tumor growth with minimal adverse side effects [148]. One limitation of this study is that monocyte-derived macrophage exosomes are not cell-specific, so the surface marker A15 was added to assist in the binding of exosomes to the malignant cells. There is another in vitro trial targeting triple-negative breast cancer, but it has used exosomes derived from natural killer cells and loaded with doxorubicin [149]. This study showed an increased rate of apoptosis and anti-angiogenesis in tumor cells when compared to free doxorubicin with a reduced toxicity to normal tissues. The limitations of this study indicate the unknown cardiotoxic effects on some in vivo models as well as lack of standardized procedure for exosome isolation and drug loading. An in vitro trial using macrophage-derived exosomes loaded with paclitaxel to target non-small cell lung cancer has demonstrated inhibition of metastases and decreased side effects compared to free paclitaxel. Subjects also had a longer survival time [150]. These exosomes also utilize surface modification to increase specificity for the lung cancer cells over normal tissue. The limitations of this study include the loading efficiency of exosomes. Mesenchymal stem-cell-derived exosomes loaded with doxorubicin have been used to target colon adenocarcinoma in vivo trials with results showing a reduced tumor growth and increased drug delivery compared to free delivery of doxorubicin [151]. The exosomes were surface modified to specifically target malignant cells. The limitations of this study include the low loading efficiency of exosomes using the electroporation method. An in vitro study using tumor-derived exosomes to deliver camptothecin in cervical cancer showed an increase in arrest of tumor cells in the S phase of the cell cycle [152]. This resulted in the tumor being more susceptible to ionizing radiation, which allowed for more tumor shrinkage without increasing the dose of radiation. There was also no evidence of systemic toxicity from exosome therapy. The limitations of this study, however, were a low loading efficiency and unknown risk in human populations. The consensuses from these disparate studies are that exosome therapies have shown potential to be effective drug delivery systems in preclinical studies, but a common problem is the cost of production that produces low yields of therapeutic exosomes. While there are not known trials for the use of exosomes in NB specifically, the early success rates in other cancers demonstrate possibility of their translation to NB treatment as well.

## 9. Challenges and Opportunities with Engineered Exosomes for Treatment of MYCN-Amplified NB

Beyond their role in disease progression, exosomes are highly attractive as natural drug delivery vehicles to MYCN-amplified NB due to several inherent advantages of exosomes [9]. Exosomes have biocompatibility and low immunogenicity. Because they are naturally secreted vesicles, exosomes are generally well-tolerated and less likely to elicit a detrimental immune response compared to synthetic nanoparticles as therapy delivery vehicles. Prudentially engineered exosomes provide stability and protection to the cargo. The lipid bilayer membrane protects their encapsulated contents (e.g., small RNA molecules, therapeutic drugs) from degradation in the bloodstream, enabling their efficient transport to the distant target sites [153]. Exosomes possess innate targeting and penetration. They can naturally home to specific cell types. In the context of MYCN-amplified NB, exosomes can be engineered or loaded with therapeutic molecules, such as small interfering RNA (siRNA), short hairpin RNA (shRNA), or tumor suppressor miRNAs, to specifically counteract the oncogenic effects of MYCN amplification. Studies have demonstrated the feasibility of using exosomes to deliver therapeutic nucleic acids. For instance, exosome-loaded siRNA targeting heat-shock protein 27 (HSP27) has been shown to induce NB cell differentiation, indicating the potential for this delivery system in a therapeutic setting [9]. Treatment with miR-186-containing exosomes secreted by activated NK cells has been shown to induce cytotoxicity in MYCN-amplified NB cells, reverse tumor-mediated immune suppression (TGF-β-induced NK cell inactivation), and reduce NB cell survival and migration [9]. This highlights the potential of using therapeutically loaded exosomes to restore the natural anti-tumor mechanisms in the body. Exosomes hold significant promise as a unique, natural-born nanocarrier system for delivering targeted therapeutics, such as tumor-suppressor miRNAs and siRNAs, and as a component of the immunotherapeutic strategies. While research is still largely preclinical, the natural properties of exosomes—including their stability, biocompatibility, and intrinsic ability to communicate with the TME—position them as a next-generation platform to overcome the aggressive nature and treatment resistance in MYCN-amplified NB. However, clinical translation will depend on advancements in exosome isolation, large-scale manufacturing, and targeted engineering for enhanced tumor specificity. Purification methods of exosomes including ultracentrifugation, size exclusion chromatography, and microfluidic separation are time intensive and costly, all of which currently impede advancement of research for using exosomes as therapeutic vehicles [154]. Also, there is no established standard yet for adequate depletion of oncogenic cargo as the use of tumor-derived exosomes remains in early preclinical stages. Current methods include loading the exosome with miRNA or siRNA to silence oncogenes [155].

Exosomes have been used in regard to management of NB in the past, but only for diagnostic or prognostic purposes in clinical settings. Exosomes from NB patients can be obtained from a blood draw and examined for DNA from tumor cells [156]. The DNA can be analyzed for mutations that are important markers for the aggressiveness of the tumor. Common mutations in high-risk NB including MYCN-amplified NB can be analyzed this way to better decide a clinical course for the patient [157]. There are ongoing studies that are evaluating the role of exosomes at the pre-clinical stage, although there have been no trials on humans at this time. There has been a study in immunotherapy using exosomes from natural killer cells cultured with NB cells [158]. These exosomes were used to target and activate other natural killer cells that had not been exposed to NB to incite a stronger immune response against the tumor cells in mice. There are also in vitro studies using tumor-derived exosomes showing successful shrinking of tumors when loaded with therapeutic nucleic acids. Exosomes are an ideal delivery system for these nucleic acids to protect them from degradation before reaching their target [159].

As mentioned earlier, one of the biggest obstacles facing NB treatment is drug resistance [160]. Tumor-derived exosomes would be a method of drug delivery that can bypass the several of the mechanisms of drug resistance in NB. They have an immunosuppressive role in several of the immune cells present in the TME, which allows them to travel to the tumor without being intercepted by immune cells [161]. They will also benefit from intrinsic recognition by the NB cell to increase uptake [162].

There have been earlier preclinical studies about the effectiveness of using siRNA to knock down MYCN expression in high-risk MYCN-amplified tumors in inhibiting tumor growth [163]. The instability of siRNA makes this difficult, but the lipid bilayer of exosomes provides the stable environment necessary for the therapeutic siRNA to be safely delivered to the tumor [164,165]. Autophagy is another method commonly used by NB to promote survival and growth. It is often activated when the cell is in distress including when the MYCN gene is knocked down [166]. Autophagy inhibitors including CQ have been used in NB to block drug resistance and suppress tumor growth [167]. Therefore, pairing the siRNA to knock down MYCN with an autophagy inhibitor will increase the efficiency of the NB treatment.

## 10. Impacts of Gut Microbiome on Treatment of MYCN-Amplified NB

Gut dysbiosis is the imbalance of microorganisms present in the gastrointestinal tract causing gut dysfunction and inflammation [168]. Emerging evidence suggests that gut dysbiosis plays a significant and bidirectional role in NB progression and response to anti-tumor treatments. A recent study involving the prospective analysis of the fecal microbiome of 15 infants (<24 months) diagnosed with NB comparatively with 17 age-matched healthy infants using 16S rRNA sequencing among other analytical methods revealed a strong dysbiosis in the gut microbiome of the NB infants [169]. Key findings included an overabundance of Pseudomonadota (synonym ‘Proteobacteria’), a major phylum of Gram-negative bacteria, ~39% in NB vs. ~12% in healthy infants with a sharp reduction in Bacteroidota (synonym ‘Bacteroidetes’) with 1.7% in NB vs. 41.4% in healthy infants. Bacteriodota are major contributors to gut health, mucosal immunity, and metabolic homeostasis. The study also revealed that standard therapy partially reversed but did not normalize the gut dysbiosis. Functional metabolism also showed gut dysbiosis-linked changes, specifically with the polyamine pathways known to promote tumor growth, support proliferation, and contribute to tumoral immune evasion. These findings provide direct evidence that NB itself is associated with significant gut dysbiosis and NB infants show a distinct pathogenic pro-inflammatory profile. Gut microbiome is thought to have a significant impact on TME, which influences how tumor cells evade immune detection [170]. Gut dysbiosis can cause increased intestinal permeability, allowing for metabolites to enter systemic circulation. This promotes the production of inflammatory cytokines including IL-6 [171]. IL-6 promotes growth and proliferation of NB cells and secretion of VEGF, a key stimulator of angiogenesis that is the process of formation of new blood vessels from the existing ones. VEGF also contributes to the metastasis of NB [172]. IL-6 can also impede exosome-based treatment of NB by activating macrophages to take up exosomes in the bloodstream [173,174]. This could cause therapeutic exosomes for NB to be absorbed before reaching the tumor itself, thereby decreasing the effectiveness of this therapeutic approach. Strategies need to be developed for clearing the inflammation in the gut for creating a calmer environment in which exosomes would have less obstacles to reach their intended target.

Another study shows a causal relationship between six different microbiota and NB in a murine model using an inverse-variance weighted method with two confirmed by meta-analysis [175]. The two confirmed microbiota were *Bifidobacterium* and *Lachnospiraceae*. Although currently there are not enough studies to establish a causal relationship between gut dysbiosis and NB in a pediatric population, there is a high correlation [176]. Most importantly, gut dysbiosis is increasingly recognized as a factor that controls the efficacy of treatments in NB, particularly in the aggressive MYCN-amplified subtype [169,176]. Studies consistently demonstrated that children with NB exhibit a state of gut dysbiosis even at the time of diagnosis before the initiation of cancer therapy [177]. This altered microbial composition is characterized by reduced richness and diversity when compared with healthy children. Specifically, NB patients have shown an abundance of certain taxa such as Pseudomonadota (including different descendants of Gammaproteobacteria and Enterobacteriaceae) and a reduced abundance of beneficial species, including butyrate producers such as *Faecalibacterium prausnitzii* and *Bifidobacterium bifidum* [178]. Furthermore, functional pathway analysis in NB patients suggested an enrichment of pathways related to protein fermentation and a reduction in carbohydrate fermentation, and a high representation of the polyamine pathway [177,178]. While these studies do not always stratify gut dysbiosis by MYCN status, the general findings establish a foundation that gut microbiome in NB patients is compromised, which is particularly relevant for high-risk MYCN-amplified group that faces the most aggressive therapeutic regimens. The oncogenic role of MYCN leads to a profound remodeling of cancer cells, influencing apoptosis resistance and the metabolic landscape, which can in turn influence the host–microbe interactions [54].

**Figure 6:**
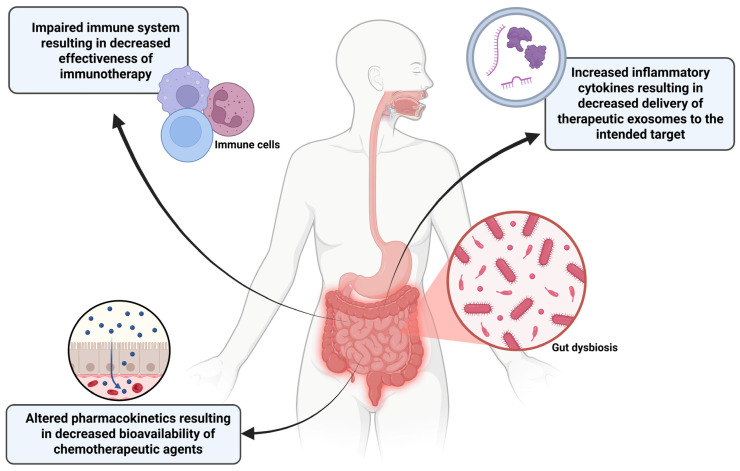
Dysbiosis of gut microbiome has multiple implications for reducing the efficacy of cancer treatments. Success rates of immunotherapy treatments can be impacted due to a diminished host immune response. Altered metabolism and absorption of chemotherapeutic drugs can decrease the dose that reaches the tumor. A dysbiotic gut increases the release of inflammatory cytokines including IL-6 that can induce macrophages in the bloodstream to take up therapeutic exosomes causing fewer to arrive at the tumor site. This figure was created using BioRender.

The standard-of-care for high-risk NB, which includes MYCN-amplified cases, involves intensive chemotherapy, surgery, radiation therapy, and immunotherapy. These treatments, especially high-dose chemotherapy, significantly disrupt the gut microbiome, often causing profound and secondary dysbiosis by reducing beneficial bacteria and increasing potentially harmful pathogens, a phenomenon that can lead to gastrointestinal side effects such as mucositis, diarrhea, and neutropenia [179]. While specific interactions with the NB chemotherapy cocktail (e.g., carboplatin, etoposide, cyclophosphamide) and the MYCN-amplified tumor are still under active investigation, it is highly probable that the altered microbial enzyme profile in gut dysbiosis can lead to altered drug pharmacokinetics, potentially resulting in sub-therapeutic drug levels at the tumor site or increased host toxicity [180]. The MYCN amplification in NB is associated with immune evasion. Gut microbiome is a key modulator of the host immune system, regulating the balance of T cell subsets (e.g., effector T cells and regulatory T cells) and influencing anti-tumor immune responses [176]. A dysbiotic gut, particularly the one lacking in butyrate-producing bacteria, can impair the ability of immune system to respond to cancer therapy, which is critical for the success of newer immunotherapeutic approaches used in high-risk NB. Conversely, certain bacteria enhance the efficacy of treatments such as immune checkpoint inhibitors (ICIs) in other cancers [181]. Given that ICIs are part of the broader arsenal for high-risk NB, gut dysbiosis seems to be a significant and unaddressed issue in treatment failure or relapse, especially in MYCN-amplified tumors, which tend to relapse within one to two years despite initial response [182]. The initial high presence of the polyamine pathway in NB patients, which is reduced following chemotherapy, suggests a functional link between the disease, the microbiome, and the specific metabolic processes [169]. Polyamines are essential for cell proliferation, and their metabolism is intricately linked to both cancer growth and the gut microbiome. In the aggressive MYCN-amplified NB subtype, which is characterized by high proliferation, this metabolic axis could represent a mechanism through which the gut microbiome supports tumor growth or controls resistance to therapies targeting tumor cell proliferation (Figure 6).

Even after standard-of-care therapy, the gut microbiome of infants with NB is only partially restored and does not fully resemble that of healthy controls. The persistence of this gut dysbiosis may contribute to long-term health issues or increase the risk of late effects of tumor treatment. For the MYCN-amplified NB patient group, which already has an inherently poor prognosis, an unresolved gut dysbiotic state poses a sustained threat to treatment success and long-term survival of the patients. The intersection of gut dysbiosis, MYCN-amplified NB, and treatment outcome represents a new frontier in pediatric oncology. The molecular aggression, driven by MYCN, coupled with a compromised and further damaged gut microbiome, creates a challenging environment for potential therapy for this tumor. Further research must focus on establishing a direct causal link between specific microbial signatures in MYCN-amplified NB and resistance to specific chemotherapeutic agents. Ultimately, identifying and targeting detrimental microbial biomarkers, possibly through probiotic, prebiotic, or fecal microbiota transplantation strategies, will offer a promising path for complementary therapeutic approaches to enhance treatment efficacy of the engineered exosome-mediated therapeutic strategy and improve the grim prognosis for children with MYCN-amplified NB.

## 11. Exosomes as Specific Delivery Vehicles of Combination Therapy to MYCN-Amplified NB and Mitigation of Gut Dysbiosis

Combination therapy is essential for high-risk NB, and novel drug delivery systems are critical for improving efficacy and reducing systemic toxicity. EVs, particularly exosomes, have emerged as promising natural nanocarriers for this purpose. Furthermore, a growing body of evidence implicates gut microbiome in NB pathogenesis and treatment response, suggesting that a successful future therapy must address the systemic effects of gut dysbiosis simultaneously [177]. The concept of using exosomes for combination therapy in MYCN-amplified NB is well-established; however, the role of gut dysbiosis in NB is still emerging. The intersection of these ideas—exosomal delivery of combination therapy to MYCN-amplified NB while specifically addressing co-existing gut dysbiosis—represents a cutting-edge and yet a highly theoretical therapeutic strategy. The inherent properties of exosomes—biocompatibility, low immunogenicity, and stability in circulation—make them ideal candidates for delivery of combination therapy. However, some preliminary efforts are necessary for engineering exosomes for drug loading. Isolated exosomes can be loaded with multiple therapeutic agents (i.e., combination therapy) through methods like electroporation or freeze–thaw cycles. For MYCN-amplified NB, an ideal combination therapy could include a small molecule inhibitor targeting the MYCN axis co-delivered with an autophagy inhibitor (e.g., CQ) and a conventional chemotherapeutic drug (e.g., vincristine) for inducing apoptosis. This multi-drug delivery system would ensure co-localization of synergistic agents at the tumor site, maximizing their combined cytotoxic effects and minimizing off-target effects.

This state of gut dysbiosis is not merely a consequence of the tumor or subsequent treatment; it is increasingly recognized as a potent systemic factor that influences the overall TME and treatment efficacy in several cancers including NB [169]. The gut–tumor axis is a bidirectional communication network primarily governed by microbial metabolites and the immune system. In MYCN-amplified NB, gut dysbiosis can affect systemic inflammation and immune evasion. An unbalanced gut microbiome can induce chronic, low-grade systemic inflammation, potentially promoting an immunosuppressive TME that favors tumor growth and metastasis. The gut microbiota can directly metabolize chemotherapeutic agents, altering their bioavailability, efficacy, and toxicity (e.g., causing treatment-related gastrointestinal side effects that reduce treatment adherence) [169]. Microbial metabolites, such as short-chain fatty acids (SCFAs), regulate host epigenetic machinery. A reduction in beneficial SCFA-producing bacteria due to gut dysbiosis can disrupt normal cellular processes and potentially fuel oncogenic pathways in distant tumors. While the engineered exosomes can deliver a combination therapy (e.g., an autophagy inhibitor and an apoptosis inducer) directly to the tumor cells, bypassing general systemic distribution, gut dysbiosis can also be addressed through other exosomes. A parallel or integrated approach could involve using microbiota-derived exosomes (MDEs) as therapeutic agents for the gut [183]. MDEs naturally arise from gut bacteria and can carry bacterial proteins and nucleic acids that modulate host immunity, intestinal barrier integrity, and local cell signaling. Alternatively, engineered exosomes could be designed to deliver a therapeutic payload to the gut mucosa to restore eubiosis [184,185]. This payload might include anti-inflammatory molecules (e.g., specific miRNAs) to repair the gut barrier and dampen systemic inflammation, promoting probiotic-derived components to encourage the growth of beneficial SCFA-producing bacteria. The use of engineered MDEs to combat dysbiosis is purely theoretical and does not have notable prior studies at this point.

The delivery of combination therapy via exosomes certainly presents a powerful strategy to overcome drug resistance in MYCN-amplified NB. Concurrently, the recognition of co-existing gut dysbiosis as a systemic factor modulating prognosis and treatment response opens a new therapeutic frontier. A highly personalized therapeutic regimen could involve two prongs: a tumor-targeted exosomal drug cocktail and a gut-targeted exosomal or MDE-based intervention to restore systemic homeostasis. While much of this combination therapeutic strategy remains purely theoretical or preclinical, the foundational science on exosomes from MYCN-amplified NB for combination therapy and for mitigating NB-associated dysbiosis provides a robust rationale for further exploring this innovative and integrative approach [177].

## 12. Conclusions and Future Directions

MYCN-amplified NB is an aggressive pediatric tumor complicated by a high rate of relapse and therapy resistance [186]. Current methods of treatment for NB include combinations of chemotherapy and immunotherapy but still have severe off-target effects and a high mortality rate in children [187]. NB tumor-derived exosomes have the potential to address these complications by increasing specificity using both natural tropism and active targeting using cell surface receptors unique to NB. Exosomes can transport small drugs, nucleic acids, and proteins [188]. Combination therapeutics loaded in exosomes have been used to treat other cancers but have not yet been applied to NB. Certainly, MYCN amplification plays a significant role in the oncogenic properties of NB and its ability to evade apoptotic cell death signals [189]. Inhibiting the expression of the MYCN gene is crucial in MYCN-amplified tumors because it is a common feature of the high-risk tumors. Autophagy is also a lucrative mechanism of drug resistance used by NB to avoid cell death by degrading damaged organelles or proteins in the cytoplasm of NB cells [190]. The NB-derived exosomes with a GPC2 target can be loaded with siRNA specific to MYCN and an effective autophagy inhibitor, CQ, will induce apoptosis in NB cells. CQ will be present to ensure that the cell does not use autophagy to survive apoptotic signals promoted by MYCN knockdown. While post-treatment relapses are still possible, this method would circumvent the known therapeutic resistance mechanisms of the relapsed NB. The above method is to specifically target MYCN-amplified NB, but it can be applied to other NB types as well. Induction of expression of tumor suppressor gene or inhibition of a potent oncogenic miRNA may provide great therapeutic effects in high-risk NB [191,192]. So, instead of using siRNA for MYCN, a tumor suppressor gene or a potent oncogenic miRNA inhibitor for promoting induction of apoptosis could be loaded along with an autophagy inhibitor in engineering therapeutic exosomes for NB treatment. If needed, exosomal therapy could be used in combination with chemotherapy or immunotherapy to increase the efficacy of the NB treatment due to weakening of drug resistance mechanism to autophagy. This can also be applied to other cancers in which an oncogene could be identified and knocked down with siRNA delivered with targeted exosomes. Addressing gut dysbiosis through use of exosomes is also an intriguing idea for future exploration and implementation in the successful treatment of MYCN-amplified NB.

## Figures and Tables

**Table 2:** Cell surface receptors on NB and targeting them for therapeutic potentials.

Receptor	Prevalence in NB	Presence in Other Tissues	Prior Trials	Reason for Therapeutic Targeting	References
GD2	Found in all NB cells primarily, low expression in relapsed tumors or during treatment	Peripheral nerve cells	Yes, results in severe side effects including neuropathic pain and low effectiveness in relapsed tumors	GD2 is a disialoganglioside present on cell surface membranes that can interact with signaling molecules for cell proliferation	[76,77,83,84]
ALK	Found in 10.9% of MYCN amplified tumors	Low levels in normal tissues	Yes, more effective in ALK-mutated tumors	ALK is a receptor tyrosine kinase known to bind on the outside of the cell for initiating a signaling cascade inside the cell	[78,85]
TrkB	High expression in MYCN amplified tumors	Found in central nervous system tissue	Yes, causes toxic side effects	TrkB is a receptor tyrosine kinase in a family of neurotrophin receptors present in neural tissue	[79,80,86]
B7-H3	High expression in MYCN amplified tumors	Low levels in normal tissues	No, but promising results in preclinical trials with CAR T cells both in vitro and in vivo	B7-H3 is a transmembrane immune checkpoint protein that interacts with immune cells	[81,87]
GPC2	90% Expression in MYCN amplified tumors	Low levels in normal tissues	No, but promising results in CAR T cell therapy in vivo trials	GPC2 is a heparan sulfate proteoglycan that triggers an internal signaling pathway	[82,88,89]

**Table 3:** Some preclinical trials conducted using exosomes as drug delivery systems.

Cancer	Exosome Origin	Agent Loaded	Model	Outcomes	Reference
Triple-negative breast cancer	A15-Exos from monocyte-derived macrophages	Doxorubicin and cholesterol modified miRNA-159	In vivo (mice)	Inhibitory effects on tumor growth	[148]
Triple-negative breast cancer	NK-Exos from natural killer cells	Doxorubicin	In vitro	Increased rate of apoptosis and anti-angiogenesis by NK-Exo delivery when compared to free delivery of doxorubicin	[149]
Non-small cell lung cancer	Macrophage-derived	Paclitaxel	In vivo (mice)	Inhibition of pulmonary metastatic growth, increased survival time, decreased side effects compared to free delivery	[150]
Colon adenocarcinoma	Mesenchymal stem cell derived	Doxorubicin	In vivo (mice)	Suppressed tumor growth, Increased drug delivery to the tumor compared to free delivery	[151]
Cervical cancer	Tumor/patient-derived	Camptothecin	In vivo (mice)	Increased tumor sensitivity to radiation by arresting the cell cycle in the S phase	[152]

## Data Availability

No new data were created or analyzed in this study. Data sharing is not applicable to this article.
